# Prognostic Nutritional Index as a Predictor of Mortality in 101,616 Patients Undergoing Hemodialysis

**DOI:** 10.3390/nu15020311

**Published:** 2023-01-08

**Authors:** Yoshikazu Miyasato, Ramy M. Hanna, Jun Morinaga, Masashi Mukoyama, Kamyar Kalantar-Zadeh

**Affiliations:** 1 Harold Simmons Center for Kidney Disease Research and Epidemiology, Division of Nephrology, Hypertension and Kidney Transplantation, School of Medicine, University of California Irvine, Orange, CA 92868, USA; 2 Department of Nephrology, Kumamoto University Graduate School of Medical Sciences, Kumamoto 860-8556, Japan

**Keywords:** hemodialysis, protein-energy wasting, mortality, albumin, lymphocyte, prognostic nutritional index

## Abstract

High mortality in dialysis patients is linked to malnutrition and inflammation. Prognostic nutritional index (PNI), calculated from serum albumin level and total lymphocyte count, has been developed as a prognostic marker for cancer patients. We investigated the clinical utility of PNI in predicting mortality in patients undergoing hemodialysis. Thus, 101,616 patients who initiated hemodialysis in United States dialysis centers between 2007 and 2011 were included in this retrospective cohort study. Using the Cox regression model, we assessed the relationship between PNI and mortality. Further, the predictive value of PNI for one-year mortality was compared with that of its constituent using area under the receiver operating characteristic curve, net reclassification improvement, and integrated discrimination improvement. Higher PNI quartiles were incrementally associated with lower mortality; in patients with PNI values of 39.5–<43.1, 43.1–<46.6, and ≥46.6 (reference: PNI < 39.5), case-mix adjusted hazard ratios (95% confidence intervals) were 0.66 (0.64, 0.68), 0.49 (0.48, 0.51), and 0.36 (0.34, 0.37), respectively. PNI predicted mortality better than serum albumin level or total lymphocyte count alone. In the subgroup analysis, PNI performed well in predicting mortality in patients aged < 65 years. Our results indicate that PNI is a simple and practical prognostic marker in patients undergoing hemodialysis.

## 1. Introduction

Protein-energy wasting (PEW) refers to the metabolic and nutritional abnormalities in patients with chronic kidney disease (CKD) and can lead to higher morbidity and mortality. Malnutrition and inflammation are important factors underlying the mechanism behind PEW, and markers of these factors are useful for the detection and management of PEW [1]. Among these markers, serum albumin level has been well studied, and patients with CKD who had low serum albumin levels showed greater mortality rates [2,3,4]. Furthermore, lymphocyte count is frequently used as an inflammatory marker [5]. In patients undergoing dialysis, low lymphocyte count was associated with poor prognosis [6,7]. These two markers are routinely checked in dialysis treatment settings and can be used for the management of PEW. Prognostic nutritional index (PNI), which is calculated from serum albumin level and total lymphocyte count, was suggested as a simple and practical screening tool for predicting prognosis in patients with cancer [8,9,10,11]. Recently, PNI has been shown to be a useful prognostic marker for diseases other than cancer, such as acute heart failure, autoimmune diseases, hematologic malignancy, and CKD in children [12,13,14,15,16].

Additionally, PNI was evaluated for its utility in predicting mortality in patients with end-stage kidney disease (ESKD); however, most studies were conducted in patients undergoing peritoneal dialysis [17,18,19]. A previous observational study evaluated the prognostic values of PNI and other inflammation-related indices in patients undergoing hemodialysis [20]. Taken together, these studies suggested that PNI could be used as a prognostic marker in patients undergoing dialysis. However, the sample sizes in these studies were relatively small, and most of these studies were conducted only in one facility. Furthermore, the utility of PNI compared with its constituents (i.e., serum albumin level or total lymphocyte count) was not investigated.

At present, the association between PNI and mortality in patients undergoing hemodialysis has not been well evaluated. Herein, we investigated the clinical utility of PNI in predicting mortality in patients undergoing hemodialysis using a large database comprising patients with ESKD and compared its utility as a prognostic marker with that of the serum albumin level and total lymphocyte count.

## 2. Materials and Methods

### 2.1. Study Population and Data Source

In this retrospective cohort study, we extracted data from a database comprising patients with ESKD who started dialysis from 1 January 2007 to 31 December 2011, in facilities of a dialysis organization in the United States (US). Follow-up term was defined from the date of the initial dialysis session in the facility until the date of death, kidney transplantation, failure to follow-up, or last follow-up evaluation. The observational time was divided into quarters and consisted of up to 20 quarters (i.e., each quarter was a 91-day period). Exclusion criteria were as follows: (1) patients who did not undergo dialysis for more than 60 consecutive days; (2) patients who received treatment modalities other than in-center hemodialysis; (3) patients who had medical histories that may affect PNI, such as cancer, autoimmune disease, liver disease, human immunodeficiency virus (HIV) infection, and transplantation; (4) patients with missing data on serum albumin level, white blood cell (WBC) count, and lymphocyte percentage at the first quarter; and (5) patients whose PNI values were in the >99.5 percentile or <0.5 percentile to minimize the likelihood of errors.

The University of California Irvine Medical Center’s Institutional Review Committee approved this study (UCI IRB# 2012-9090, approval on 21 March 2013). Due to the large sample size, maintenance of patients’ anonymity, and the nonintrusive nature of the study, the need for informed consent was waived.

### 2.2. Clinical, Demographic, and Laboratory Data

From the database, we extracted the data of age, sex, race, ethnicity, death information, comorbidities, medical insurance, type of vascular access, height, weight, and laboratory data. Comorbidities (e.g., diabetes, hypertension, dyslipidemia, atherosclerotic heart disease (ASHD), congestive heart failure (CHF), other cardiovascular disease (CVD), chronic obstructive pulmonary disease (COPD), and substance abuse) were defined referring to the ICD9 codes in the database. Most laboratory data were recorded on a monthly basis. Some patients had their hemoglobin levels checked monthly, while most patients had them checked weekly or biweekly. Ferritin and intact parathyroid hormone (iPTH) were assessed on a quarterly basis. To reduce measurement variability, repeated laboratory data were averaged within each quarter, and data from the first quarter were used as baseline data. All dialysis facilities used the same procedures for drawing blood samples, which were sent to the main laboratory in Deland, Florida. Automated and standardized methods were used to measure all laboratory data. Most blood specimens were collected prior to the dialysis session except for post-dialysis serum urea nitrogen to calculate urea kinetic parameters, such as normalized protein catabolic rate (nPCR) and single-pool Kt/V (spKt/V). Total neutrophil count was calculated using the following formula: [WBC count (/mm^3^)] × [neutrophil percentage/100]. Total lymphocyte count was calculated using the following formula: [WBC count (/mm^3^)] × [lymphocyte percentage/100]. The following formula was used to compute PNI: [10 × serum albumin (g/dL)] + [0.005 × total lymphocyte count (/mm^3^)] [8]. Body mass index (BMI) was calculated by dividing post-hemodialysis dry weight by baseline height squared (kg/m^2^). Geriatric nutritional risk index (GNRI) was calculated by [14.89 × serum albumin (g/dL)] + [41.7 × BMI (kg/m^2^)/22] [21]. Neutrophil to lymphocyte ratio (NLR) was calculated by dividing total neutrophil count (/mm^3^) by total lymphocyte count (/mm^3^) [5].

### 2.3. Outcome Ascertainment

The primary outcomes were all-cause and cardiovascular (CV) deaths, which were retrieved from the database. CV death included death from myocardial infarction, CHF, ASHD, valvular heart disease, cardiac arrhythmia, pulmonary edema, cardiomyopathy, pulmonary embolus, pericarditis, cardiac arrest, cerebrovascular accident, anoxic encephalopathy, and ischemic brain damage.

### 2.4. Statistical Analysis

The participants were separated into four groups based on the PNI quartiles, which were rounded to the first decimal place. Summary statistics of the baseline data were calculated for all subjects and the four PNI groups. For continuous variables, means and standard deviations or medians and interquartile ranges were used to express, whereas percentages were used for discrete variables. We used a non-parametric trend test to assess the relationship between the four PNI groups and patients’ characteristics.

We estimated the survival rates of the PNI groups using Kaplan–Meier analysis, and the differences in the survival estimates were tested using the log-rank test. The participants were followed up and considered at risk until death or censoring for kidney transplantation, transfer to other dialysis facilities, or the end of the study period (31 December 2011). For evaluating the relationship between PNI and all-cause or CV mortalities, we used Cox regression models, which included three-level-adjusted models: (1) unadjusted model, (2) case-mix-adjusted model, and (3) malnutrition–inflammation complex syndrome (MICS)-adjusted model. The variables examined in these models were based on those of previous studies [3,5]. (1) The unadjusted model consisted of PNI. (2) The case-mix-adjusted model included PNI and the following variables: age, sex, race, ethnicity, medical insurance, type of vascular access, comorbidities (e.g., diabetes, hypertension, dyslipidemia, ASHD, CHF, other CVD, COPD, and substance abuse), nPCR, spKt/V, and natural log-transformed BMI. (3) The MICS-adjusted model included all the variables in the case-mix-adjusted model, WBC count, hemoglobin, creatinine, serum albumin, albumin-corrected calcium, phosphorus, bicarbonate, iron saturation, total iron-binding capacity (TIBC), and natural log-transformed iPTH, alkaline phosphatase (ALP), and ferritin. Additionally, we used time-varying Cox regression models to evaluate the relationship between PNI and all-cause or CV mortalities in each 91-day period. In the time-varying models, PNI, type of vascular access, nPCR, spKt/V, WBC count, hemoglobin, creatinine, serum albumin, albumin-corrected calcium, phosphorus, bicarbonate, iron saturation, TIBC, and natural log-transformed BMI, iPTH, ALP, and ferritin in each 91-day period were used as time-varying covariates, while age, sex, race, ethnicity, medical insurance, and comorbidities in the initial 91-day period were used as baseline covariates.

To compare the mortality predictability of PNI, serum albumin level, and total lymphocyte count, we evaluated the area under the receiver operating characteristic curve (AUROC), continuous net reclassification improvement (NRI), and integrated discrimination improvement (IDI) for one-year all-cause mortality using logistic regression analysis with case-mix-adjusting variables. We also conducted the same analysis by setting the cut-off values of PNI as 35, 40, or 45, which were used in previous studies [9,17,20,22,23] or by using GNRI and NLR.

We performed subgroup analyses with a case-mix-adjusted Cox regression model and interaction tests stratified by age (<65 and ≥65), sex (male and female), and BMI (<18.5, 18.5–<25, 25–<30, and ≥30). In these analyses, we compared the patients in the first quartile or with higher PNI (PNI ≥ 39.5) to those with PNI less than the first quartile (PNI < 39.5). The AUROC, NRI, and IDI for one-year all-cause mortality were also evaluated in the groups stratified by age.

The frequencies of missing data were 1.5% for nPCR, 4.6% for creatinine, 1.2% for ferritin, and <1% for BMI, spKt/V, and other laboratory data. Missing baseline data were imputed using the multiple-imputation method, in which each missing value was replaced with a substituted value using the following variables: age, sex, race, ethnicity, medical insurance, type of vascular access, and comorbidities (e.g., diabetes, hypertension, dyslipidemia, ASHD, CHF, other CVD, COPD, and substance abuse). We created five filled-in complete datasets and then pooled the parameter estimates using Rubin’s rule [24]. Missing data from the 2nd to 20th periods were imputed using the last-observation-carried-forward method for time-varying analysis.

Statistical significance was set as *p* < 0.05. Most analyses were conducted with STATA MP version 13.1 (Stata Corp, College Station, TX, USA). The AUROC, NRI, and IDI were calculated using SAS 9.4 (SAS Institute, Cary, NC, USA).

## 3. Results

### 3.1. Characteristics of the Study Population

**Figure 1:**
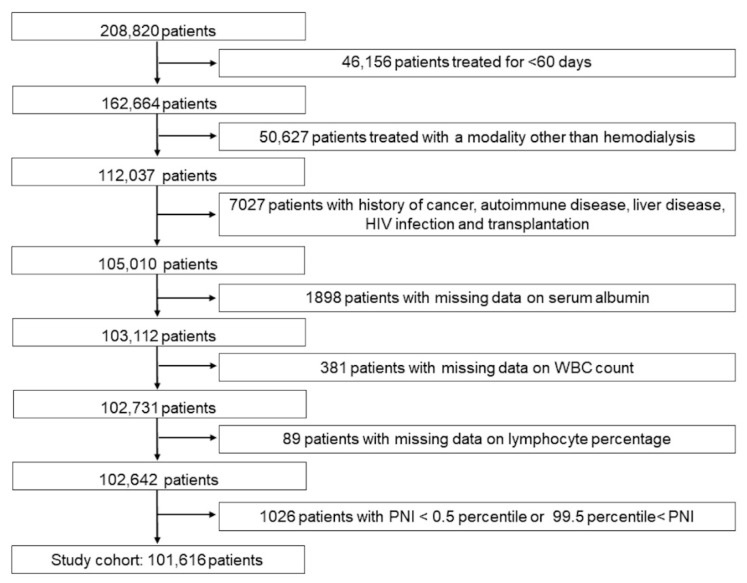
Flowchart of patient selection in the study cohort. Abbreviations: PNI, prognostic nutritional index; HIV, human immunodeficiency virus; WBC, white blood cell.

Of the 208,820 patients, we excluded the following patients: 46,156 patients who did not undergo dialysis for more than 60 consecutive days; 50,627 patients who received treatment modalities other than hemodialysis; 7027 patients who had a history of cancer, autoimmune disease, liver disease, HIV infection, or transplantation; 2368 patients with missing data on serum albumin level, WBC count, and lymphocyte percentage at the first quarter; and 1026 patients whose PNI values were in the >99.5 percentile or <0.5 percentile. The final cohort comprised 101,616 patients (Figure 1).

The final cohort’s demographics included a mean age of 63 ± 15 years, 56% male, 46% Caucasian, 15% Hispanic, and a mean PNI value of 42.9 ± 5.4. Baseline characteristics were compared between the four quartiles of PNI (<39.5, 39.5–<43.1, 43.1–<46.6, and ≥46.6). Patients with higher PNI tended to be younger, male, and non-Caucasian. They also showed less central venous catheter use; higher BMI and prevalence of hypertension; lower prevalence of diabetes and CHF; higher levels of hemoglobin, creatinine, and phosphorus; and lower levels of ferritin and bicarbonate (Table 1).

### 3.2. Association between Prognostic Nutritional Index and Mortality

**Figure 2:**
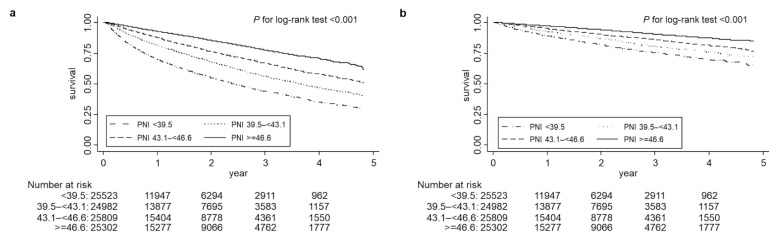
Kaplan–Meier survival plots between quartiles of prognostic nutritional index (PNI) for all-cause death (**a**) and cardiovascular death (**b**) in the entire cohort of 101,616 patients undergoing hemodialysis. Survival differences were compared using the log-rank test. Number at risk in each group divided by quartiles of PNI is shown below the plots. Abbreviations: PNI, prognostic nutritional index.

**Figure 3:**
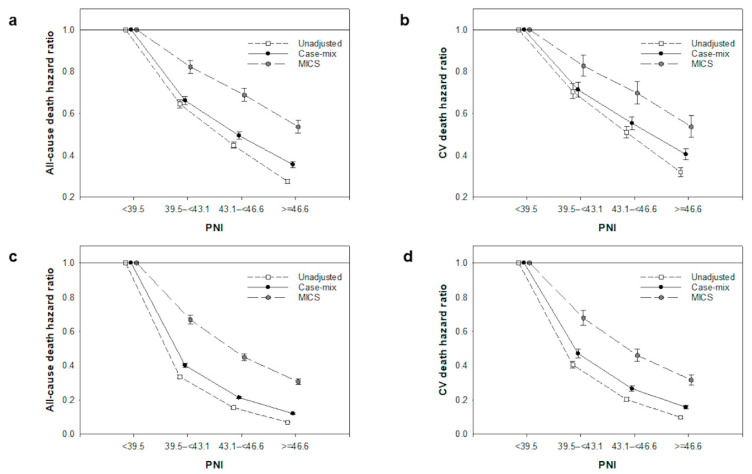
Association of prognostic nutritional index (PNI) with all-cause and cardiovascular mortalities using the Cox regression model adjusted for baseline variables (**a**,**b**) and time-varying variables (**c**,**d**) in the entire cohort of 101,616 patients undergoing hemodialysis. Hazard ratios for mortality in the Cox regression models with three-level adjustments are compared between quartiles of PNI (reference: PNI < 39.5). Error bars show 95% confidence intervals of hazard ratios. Abbreviations: PNI, prognostic nutritional index; MICS, malnutrition–inflammation complex syndrome; CV, cardiovascular.

During the median 1.4-years of follow-up, 26,622 patients (26%) died among the 101,616 participants. CV death comprised 36% of the total deaths (n = 9659). The follow-up period of each reason for censoring and proportions of other causes of death are shown in Supplementary Table S1 and Supplementary Figure S1. Both all-cause and CV deaths decreased with increasing quartiles of PNI (Figure 2a,b). Higher PNI quartiles were linked to lower mortality in the Cox regression models that were unadjusted and adjusted for case-mix and MICS variables. Among the patients with PNI of 39.5–<43.1, 43.1–<46.6 and ≥46.6 (reference: PNI < 39.5), the case-mix-adjusted hazard ratios (HRs) (95% confidence intervals (CIs)) for all-cause mortality were 0.66 (0.64, 0.68), 0.49 (0.48, 0.51), and 0.36 (0.34, 0.37), respectively, and those for CV mortality were 0.71 (0.68, 0.75), 0.55 (0.52, 0.58), and 0.40 (0.38, 0.43), respectively (Figure 3a,b). Similar trends were observed when the MICS variables were adjusted (Figure 3a,b). In the time-varying covariate model, higher PNI was also associated with lower mortality (Figure 3c,d).

### 3.3. Comparison between Prognostic Nutritional Index and Its Constituents in Predicting Mortality

To compare the mortality predictability of PNI, serum albumin level and total lymphocyte count, we calculated the AUROC, NRI, and IDI of these parameters for one-year all-cause mortality. PNI showed higher values for the AUROC, NRI, and IDI than serum albumin level and total lymphocyte count in the case-mix-adjusted logistic regression models, and differences in ∆AUROC between PNI and its constituents were found to be statistically significant (Table 2).

### 3.4. Subgroup Analysis

**Figure 4:**
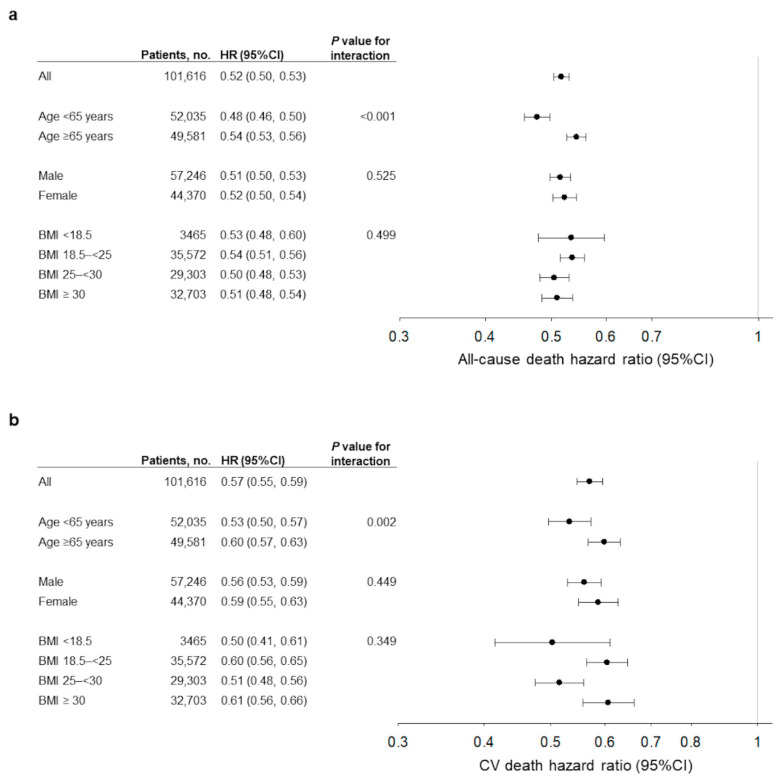
Association of prognostic nutritional index (PNI) with all-cause (**a**) and cardiovascular mortalities (**b**) in the subgroup analysis. Hazard ratios for mortality in the case-mix-adjusted Cox regression models are compared between PNI ≥ 39.5 and PNI < 39.5 (reference). Error bars show 95% confidence intervals of hazard ratios. p values for interaction between each subgroup variable and PNI are shown. Abbreviations: HR, hazard ratio; CI, confidence interval; CV, cardiovascular; BMI, body mass index.

When we stratified the age (<65 and ≥65), sex (male and female), and BMI (<18.5, 18.5–<25, 25–<30, and ≥30) in the subgroup analysis, higher PNI (≥39.5, reference: <39.5) was consistently associated with lower case-mix-adjusted HRs for all-cause and CV mortalities. A statistically significant interaction was found in the age groups (Figure 4a,b). In the subgroup analysis for age, the younger group showed lower HRs than the older group (Figure 4a,b). Although PNI showed higher AUROC, NRI, and IDI values for one-year all-cause mortality than serum albumin level and total lymphocyte count in the younger group, there was no significant difference in ∆AUROC between PNI and serum albumin level in the older group (Table 3).

## 4. Discussion

In this historical cohort study, we assessed the association between PNI and mortality using a nationally representative cohort of 101,616 patients undergoing hemodialysis. Higher PNI was associated with decreased all-cause and CV mortalities, and the association was consistent across subgroups. We also showed that PNI is a better predictor of mortality than serum albumin level and total lymphocyte count, especially in patients aged < 65 years.

Previous observational studies have shown that higher PNI is linked to lower mortality in patients undergoing dialysis [17,18,19,20]. However, these studies had some limitations: the sample sizes were small and the studies were conducted in only one or two facilities. Herein, we used the database of a large US dialysis organization and demonstrated the consistent inverse association between PNI and mortality. Compared with previous studies, our analyses were derived from a larger sample size and multiple facilities and explored various variables. We also assessed the relationship between PNI and outcomes using baseline and time-dependent variables and found significant associations between PNI and these outcomes. Furthermore, we evaluated the effects of PNI, serum albumin level, and total lymphocyte count on mortality prediction models by calculating the AUROC, NRI, and IDI.

To date, many prognostic markers related to inflammation and nutrition have been reported [5,9,25,26]. A previous observational study evaluated the association between six inflammation-based prognostic scores (i.e., PNI, Glasgow prognostic score (GPS), modified GPS, prognostic index, NLR, and platelet to lymphocyte ratio) and mortality in patients undergoing hemodialysis, revealing statistically significant AUROC for overall mortality in PNI and GPS [20]. C-reactive protein (CRP) and serum albumin level are used for calculating GPS. Several studies have shown that GPS has higher prognostic power than PNI in patients undergoing dialysis; however, its application in current routine clinical practice is limited due to cost and specific clinical situations for measuring CRP (e.g., active infection status) [17,20]. In contrast, the total lymphocyte count, which is one of the constituents of PNI, is routinely measured in clinical settings. In our study, we could not compare the predictive values of PNI with those of GPS for mortality, because CRP was measured sporadically in a small number of patients. Among other prognostic markers related to inflammation and nutrition, NLR was examined for its utility for mortality predictability using the same dialysis database as this study previously [5], and GNRI, calculated using serum albumin and BMI, is another nutritional index well examined with mortality [21]. Therefore, we compared mortality predictability between PNI, GNRI, and NLR and found that PNI predicts mortality better (Supplementary Table S2). We believe that detecting patients at risk of poor prognosis using routinely measured markers and performing more detailed evaluations and interventions, as needed, are significant. Thus, PNI has the potential to serve as a practical prognostic marker in daily clinical settings.

In terms of the clinical implementation of PNI, there may be some conditions to consider. Although the serum albumin level has been well investigated and established as a marker of MICS for mortality prediction [3], the level of serum albumin was shown to be associated with fluid overload as well as malnutrition and inflammation in patients undergoing dialysis [27,28]. Therefore, the utility of serum albumin level as a prognostic marker has been controversial in the early period of dialysis [19]. However, Kang et al. showed that PNI is independently related to mortality by adjusting the fluid status in patients undergoing peritoneal dialysis (PD), which suggested that PNI may be a practical prognostic marker, even in the early stage of dialysis [19]. In this study, we further confirmed that PNI was associated with mortality in a time-varying covariate model, indicating that PNI could serve as a prognostic marker in any stage of dialysis.

In our subgroup analysis stratified by age, sex, and BMI, PNI was independently associated with mortality. When we stratified age by 65 years, PNI was a better predictor of mortality in the younger group than that of the older group, and a significant difference in AUROC between PNI and serum albumin was observed in the younger group, but not in the older group. Valiathan et al. showed that lymphocyte count decreases with increasing age [29]. Furthermore, a previous study reported that lymphocyte count is not an appropriate nutritional marker for older patients because it reflects their age rather than their nutritional status [30]. Thus, the influence of age on lymphocyte count could explain why no AUROC difference between PNI and serum albumin was observed in patients aged ≥ 65 years. Our results suggested that the clinical utility of PNI as a prognostic marker was superior to that of the serum albumin level in patients aged < 65 years undergoing hemodialysis, although its clinical utility is comparable to that of serum albumin level in patients aged ≥ 65 years.

Furthermore, we showed the association between PNI and mortality using PNI quartiles. Although PNI was widely examined as a prognostic marker in various diseases, the cut-off value of PNI has not been defined. Previous studies used 35, 40, or 45 as the cut-off points for PNI [9,17,20,22,23]. We assessed the predictability of PNI for mortality in our model using these three cut-off points, and a cut-off point of 40 appeared to be the optimal value in our research population (Supplementary Table S3). In terms of its clinical utility, it may be necessary to establish the ideal cut-off value of PNI for clinical decision making, and further investigation is required to identify the cut-off point of PNI as a prognostic marker.

There were some limitations in this study. First, because our study was observational, we were unable to elucidate the causation between PNI and mortality. We also could not exclude the possibility of residual confounding effects due to unmeasured confounders. Second, we could not obtain information about the infection and fluid status of patients, which could affect the serum albumin level and total lymphocyte count. Third, other models, including different variables from our models, may predict mortality better in patients undergoing hemodialysis because there were significant associations between PNI quartiles and many variables in the trend test. Fourth, we did not compare the prediction power of PNI for mortality with that of more complex nutritional indicators, including malnutrition–inflammation score, because the specific scoring data could not be obtained from the database [31].

In summary, using a large cohort from a US dialysis organization, we demonstrated that higher PNI was associated with decreased mortality in patients undergoing hemodialysis. Moreover, PNI was a better predictor for one-year mortality than its constituents (i.e., serum albumin and total lymphocyte count), especially in patients aged < 65 years. PNI can be easily evaluated in daily clinical settings, as the constituents of PNI are common laboratory data. Therefore, our results support the use of PNI as a simple and practical prognostic marker in patients undergoing hemodialysis.

## Figures and Tables

**Table 1:** Baseline characteristics of 101,616 patients stratified by quartiles of prognostic nutritional index.

	Total	Prognostic Nutritional Index				
		<39.5	39.5–<43.1	43.1–<46.6	≥46.6	
	n = 101,616	n = 25,523	n = 24,982	n = 25,809	n = 25,302	*p* for Trend
Age (Mean ± SD)	63 ± 15	65 ± 15	65 ± 14	63 ± 14	59 ± 16	<0.001
Male, %	56	55	56	57	58	<0.001
Race and Ethnicity, %						<0.001
Caucasian	46	50	49	45	41	
African-American	31	28	29	33	35	
Hispanic	15	15	15	15	17	
Asian	3.3	3.2	3.3	3.3	3.6	
Other	3.7	3.9	3.6	3.6	3.6	
Medical insurance, %						<0.001
Medicare	54	57	57	53	48	
Medicaid	7.0	7.2	6.1	6.7	8.0	
Other	39	36	37	40	44	
Type of vascular access, %						<0.001
CV catheter	75	82	77	72	67	
AV fistula/graft	19	12	17	22	26	
Unknown	5.9	5.7	5.5	6.0	6.6	
Body mass index, kg/m^2^	26.7 (23.0–31.8)	25.5 (22.2–30.4)	26.6 (23.0–31.7)	27.2 (23.4–32.3)	27.6 (23.7–32.7)	<0.001
Single-pool Kt/V (Mean ± SD)	1.47 ± 0.32	1.43 ± 0.32	1.47 ± 0.32	1.48 ± 0.32	1.48 ± 0.33	<0.001
nPCR, g/kg per day (Mean ± SD)	0.79 ± 0.22	0.73 ± 0.21	0.79 ± 0.21	0.82 ± 0.21	0.84 ± 0.21	<0.001
Comobidities, %						
Diabetes	59	63	63	59	50	<0.001
Hypertension	50	45	49	51	55	<0.001
Dyslipidemia	24	23	24	25	24	0.009
Atherosclerotic heart disease	14	14	15	14	12	<0.001
Congestive heart failure	36	39	38	35	32	<0.001
Other cardiac disease	14	15	15	14	12	<0.001
COPD	4.0	4.5	4.3	3.9	3.3	<0.001
Alcohol	0.2	0.2	0.2	0.2	0.2	0.504
Substance abuse	0.2	0.2	0.2	0.2	0.3	0.008
Laboratory variables						
Hemoglobin, g/dL (Mean ± SD)	11.1 ± 1.2	10.7 ± 1.2	11.1 ± 1.1	11.3 ± 1.1	11.5 ± 1.1	<0.001
WBC, 10^3^/mm^3^	7.5 (6.2–9.1)	7.4 (5.9–9.1)	7.3 (6.0–8.8)	7.4 (6.2–8.9)	7.8 (6.6–9.3)	<0.001
Total neutrophil, 10^3^/mm^3^	5.1 (4.0–6.4)	5.3 (4.1–6.9)	5.1 (4.0–6.4)	5.0 (4.0–6.2)	5.0 (4.0–6.2)	<0.001
Total lymphocyte, 10^3^/mm^3^	1.5 (1.1–1.9)	1.2 (0.9–1.5)	1.3 (1.1–1.6)	1.5 (1.2–1.8)	1.9 (1.6–2.3)	<0.001
Serum albumin, g/dL (Mean ± SD)	3.5 ± 0.5	3.0 ± 0.4	3.4 ± 0.2	3.7 ± 0.2	4.0 ± 0.3	<0.001
Creatinine, mg/dL (Mean ± SD)	5.9 ± 2.4	5.1 ± 1.9	5.5 ± 2.1	6.0 ± 2.3	6.8 ± 2.7	<0.001
Calcium, mg/dL (Mean ± SD)	9.1 ± 0.6	9.1 ± 0.6	9.1 ± 0.6	9.1 ± 0.6	9.1 ± 0.6	0.002
Phosphorus, mg/dL (Mean ± SD)	4.9 ± 1.1	4.7 ± 1.2	4.9 ± 1.1	5.0 ± 1.1	5.1 ± 1.2	<0.001
Intact PTH, pg/mL	315 (198–487)	273 (168–419)	310 (197–476)	331 (213–509)	352 (223–548)	<0.001
Alkaline phosphatase, IU/L	87 (69–115)	97 (75–134)	88 (70–116)	84 (67–108)	81 (65–104)	<0.001
TIBC, μg/dL (Mean ± SD)	226.0 ± 48.4	194.4 ± 46.5	223.5 ± 43.3	236.6 ± 42.4	249.4 ± 43.1	<0.001
Iron saturation, % (Mean ± SD)	22.9 ± 8.8	23.4 ± 10.3	22.3 ± 8.3	22.5 ± 8.0	23.3 ± 8.2	<0.001
Ferritin, ng/mL	279 (162–476)	332 (188–588)	279 (162–476)	262 (155–443)	252 (149–418)	<0.001
Bicarbonate, mEq/L (Mean ± SD)	23.6 ± 2.7	24.1 ± 2.8	23.8 ± 2.6	23.5 ± 2.6	23.0 ± 2.6	<0.001

Continuous variables are expressed as mean ± standard deviation (SD) or median (Interquartile range (IQR)). Discrete variables are expressed as percentages. Data were compared between groups using a non-parametric trend test. Abbreviations: CV, central venous; AV, arteriovenous; nPCR, normalized protein catabolic rate; COPD, chronic obstructive pulmonary disease; WBC, white blood cell; PTH, parathyroid hormone; TIBC, total iron-binding capacity.

**Table 2:** Analyses of the area under the receiver operating characteristic curve, net reclassification improvement, and integrated discrimination improvement for one-year all-cause mortality by including the prognostic nutritional index, serum albumin level, or total lymphocyte count in the case-mix-adjusted logistic regression model.

	AUROC (95%CI)	∆AUROC (95%CI)	NRI (95%CI)	IDI (95%CI)
PNI	0.747 (0.743, 0.751)	Reference	0.434 (0.416, 0.452)	0.033 (0.032, 0.035)
Serum albumin (g/dL)	0.744 (0.740, 0.749)	−0.003 (−0.005, −0.001)	0.409 (0.390, 0.427)	0.031 (0.030, 0.032)
Total lymphocyte count (10^3^/mm^3^)	0.713 (0.708, 0.717)	−0.034 (−0.037, −0.032)	0.174 (0.155, 0.192)	0.003 (0.003, 0.004)

PNI, serum albumin, and total lymphocyte count were used as continuous variables. Case-mix variables were age, sex, race, ethnicity, medical insurance, type of vascular access, comorbidities (diabetes, hypertension, dyslipidemia, arteriosclerotic heart disease, chronic heart failure, other cardiovascular disease, chronic obstructive pulmonary disease, substance abuse), single pool Kt/V, normalized protein catabolic rate, and natural log-transformed BMI. Abbreviations: AUROC, area under the receiver operating characteristic curve; NRI, net reclassification improvement; IDI, integrated discrimination improvement; CI, confidence interval.

**Table 3:** Age-stratified analyses of the area under the receiver operating characteristic curve, net reclassification improvement, and integrated discrimination improvement for one-year all-cause mortality by including the prognostic nutritional index, serum albumin level, or total lymphocyte count in the case-mix-adjusted logistic regression model.

	AUROC (95%CI)	∆AUROC (95%CI)	NRI (95%CI)	IDI (95%CI)
Age < 65 years				
PNI	0.731 (0.723, 0.739)	Reference	0.479 (0.448, 0.511)	0.032 (0.030, 0.034)
Serum albumin (g/dL)	0.723 (0.715, 0.731)	−0.008 (−0.011, −0.005)	0.421 (0.389, 0.453)	0.026 (0.024, 0.028)
Total lymphocyte count (10^3^/mm^3^)	0.685 (0.676, 0.693)	−0.047 (−0.052, −0.041)	0.207 (0.175, 0.239)	0.005 (0.004, 0.006)
Age ≥ 65 years				
PNI	0.692 (0.686, 0.698)	Reference	0.392 (0.370, 0.415)	0.037 (0.035, 0.038)
Serum albumin (g/dL)	0.691 (0.685, 0.697)	−0.002 (−0.004, 0.001)	0.379 (0.357, 0.402)	0.035 (0.033, 0.037)
Total lymphocyte count (10^3^/mm^3^)	0.644 (0.638, 0.651)	−0.048 (−0.052, −0.044)	0.148 (0.126, 0.171)	0.003 (0.003, 0.004)

PNI, serum albumin, and total lymphocyte count were used as continuous variables. Case-mix variables were age, sex, race, ethnicity, medical insurance, type of vascular access, comorbidities (diabetes, hypertension, dyslipidemia, arteriosclerotic heart disease, chronic heart failure, other cardiovascular disease, chronic obstructive pulmonary disease, substance abuse), single pool Kt/V, normalized protein catabolic rate, and natural log-transformed BMI. Abbreviations: AUROC, area under the receiver operating characteristic curve; NRI, net reclassification improvement; IDI, integrated discrimination improvement; CI, confidence interval.

## Data Availability

Due to the nature of this research, participants of this study did not agree for their data to be shared publicly, so supporting data are not available.

## Supplementary Materials

The following supporting information can be downloaded at: https://www.mdpi.com/article/10.3390/nu15020311/s1, Table S1: The follow-up period of each reason for censoring, Table S2: Analyses of the area under the receiver operating characteristic curve, net reclassification improvement, and integrated discrimination improvement for one-year all-cause mortality by adding PNI, GNRI, or NLR to the case-mix-adjusted logistic regression model, Table S3: Analyses of the area under the receiver operating characteristic curve, net reclassification improvement, and integrated discrimination improvement for one-year all-cause mortality using the three cut-off values of prognostic nutritional index in the case-mix-adjusted logistic regression model, Figure S1: The causes of death in 26,622 deaths of the hemodialysis cohort.
