# Supplementary material for: Prognostic Nutritional Index as a Predictor of Mortality in 101,616 Patients Undergoing Hemodialysis
Source: Nutrients. 2023 Jan 8;15(2):311. doi: 10.3390/nu15020311 (PMC9865495; doi:10.3390/nu15020311)

Supplementary table S1. The follow-up period of each reason for censoring

Reasons for censoring	Median (year)	IQR	(%)
Death	1.05	(0.53, 1.93)	26.20
Discharge to other dialysis facility	0.85	(0.44, 1.63)	5.79
Kidney transplantation	1.37	(0.75, 2.26)	3.50
Discontinued dialysis	0.65	(0.35, 1.22)	1.99
Regained renal function	0.37	(0.26, 0.68)	1.98
Missing/Other	1.71	(0.81, 2.92)	60.54
Total	1.36	(0.64, 2.52)	100.00

Abbreviations: IQR, Interquartile range

Supplementary table S2. Analyses of the area under the receiver operating characteristic curve, net reclassification improvement, and integrated discrimination improvement for one-year all-cause mortality by adding PNI, GNRI, or NLR to the case-mix adjusted logistic regression model

	AUROC (95%CI)	Δ AUROC (95%CI)	NRI (95%CI)	IDI (95%CI)
PNI	0.747 (0.743, 0.751)	Reference	0.434 (0.416, 0.452)	0.033 (0.032, 0.035)
GNRI	0.737 (0.732, 0.741)	−0.010 (−0.012, −0.008)	0.377 (0.359, 0.396)	0.024 (0.022, 0.025)
NLR	0.726 (0.722, 0.731)	−0.021 (−0.023, −0.018)	0.297 (0.279, 0.316)	0.014 (0.012, 0.015)

PNI, GNRI, and NLR were used as continuous variables.
Case-mix variables were age, sex, race, medical insurance, vascular access type, comorbidities (hypertension, diabetes, dyslipidemia, arteriosclerotic heart disease, chronic heart failure, other cardiovascular disease, chronic obstructive pulmonary disease, substance abuse), single pool Kt/V, normalized protein catabolic rate, and natural log-transformed BMI.
Abbreviations: AUROC, area under the receiver operating characteristic curve; NRI, net reclassification improvement; IDI, integrated discrimination improvement; CI, confidence interval; Ref, reference; PNI, prognostic nutritional index; GNRI, geriatric nutritional risk index; NLR, neutrophil to lymphocyte ratio

Supplementary table S3. Analyses of the area under the receiver operating characteristic curve, net reclassification improvement, and integrated discrimination improvement for one-year all-cause mortality using the three cut-off values of prognostic nutritional index in the case-mix adjusted logistic regression model

	AUROC (95%CI)	Δ AUROC (95%CI)	NRI (95%CI)	IDI (95%CI)
PNI ≥35 (Ref: <35)	0.725 (0.721, 0.730)	Reference	0.169 (0.154, 0.185)	0.017 (0.016, 0.018)
PNI ≥40 (Ref: <40)	0.735 (0.731, 0.740)	0.010 (0.008, 0.013)	0.476 (0.458, 0.494)	0.022 (0.021, 0.023)
PNI ≥45 (Ref: <45)	0.729 (0.725, 0.733)	0.004 (0.001, 0.007)	0.444 (0.430, 0.459)	0.013 (0.013, 0.014)

Case-mix variables were age, sex, race, medical insurance, vascular access type, comorbidities (hypertension, diabetes, dyslipidemia, arteriosclerotic heart disease, chronic heart failure, other cardiovascular disease, chronic obstructive pulmonary disease, substance abuse), single pool Kt/V, normalized protein catabolic rate, and natural log-transformed BMI.

Abbreviations: AUROC, area under the receiver operating characteristic curve; NRI, net reclassification improvement; IDI, integrated discrimination improvement; CI, confidence interval; Ref, reference

Supplementary figure S1. The causes of death in 26,622 deaths of the hemodialysis cohort

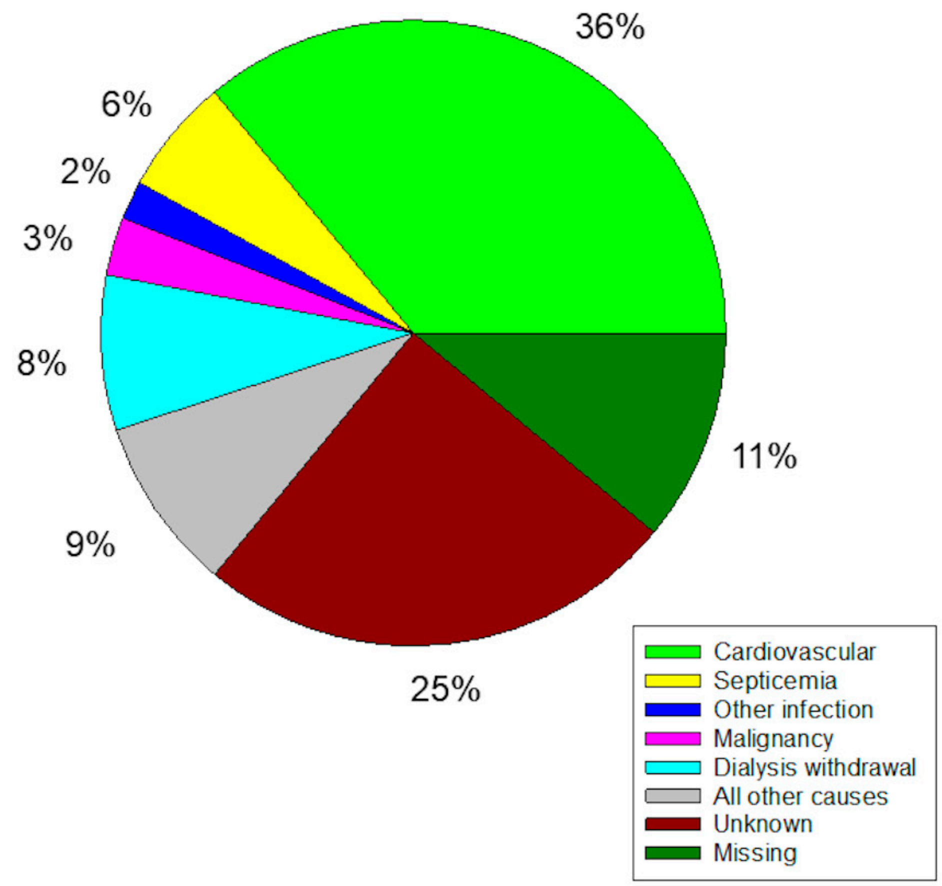

Supplement: Supplementary file 1 [file nutrients-15-00311-s001.zip › nutrients-2111569-supplementary.pdf]
